# Technical implications of new IUPAC elements in cheminformatics

**DOI:** 10.1186/s13321-017-0196-0

**Published:** 2017-02-13

**Authors:** John W. Mayfield, Roger A. Sayle

**Affiliations:** NextMove Software Ltd, Innovation Centre (Unit 23), Science Park, Cambridge, CB4 0EY UK

**Keywords:** IUPAC, Elements, Periodic, Cheminformatics, SMARTS

## Abstract

The symbols for the new IUPAC elements named in November 2016 can introduce subtle ambiguities within cheminformatics software. The ambiguities are described and demonstrated by highlighting inconsistencies between software when handling existing element symbols.

## Background

On the 28th November 2016 the International Union of Pure and Applied Chemistry (IUPAC) approved the names and symbols of four new elements: 113 Nihonium (**Nh**), 115 Moscovium (**Mc**), 117 Tennessine (**Ts**), 118 Oganesson (**Og**). Cheminformatics libraries typically use a centralised dictionary of elements to store and look up symbols in the periodic table. Naïvely adding the new element symbols to this table can introduce unexpected behaviour.

## Contracted abbreviations

**Fig. 1:**
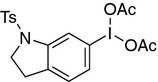
6-(Diacetoxyiodo)-1-tosylindoline Intermediate 30 in US 2016/362375 A1. The intended meaning of **Ts** is Tosyl, and OAc is Acetoxy

The first ambiguity was noted in the the preliminary recommendations from IUPAC. Contracted group abbreviations are common in published chemistry research to make sketches more concise and readable. Common abbreviations include Ph for Phenyl, NO_2_ for Nitro, COOH for Carboxylic acid, etc. The new symbol **Ts** is widely used to represent a Tosyl group. Unfortunately the symbol Tn could not be used as it was previously used for $^{220}$Radon (Thoron). It was noted in the preliminary recommendations that similarly to how Ac is used for both Actinium and Acetyl (and sometimes Acyl) the intended meaning would be clear from the context (Fig. 1).

While it is true that a human may decipher the intended meaning, it is more difficult for the software especially when the compound is no longer associated with the original context. The existence of compounds like PubChem [1]’s *p*-tolylactinium (CID 20712520 [2]) instead of the intended *p*-acetyl structure demonstrate this. The error here was propagated from the original substance submissions: a deprecated ChemSpider [3] entry, and patent sketches extracted by SCRIPDB [4].

Software for sketching chemical diagrams often allow the input of contracted abbreviations. ChemDraw 15 interprets all Ac labels as Acetyl and it is impossible to add an Actinium atom to a sketch even via the periodic table selection menu or from file or line-notation input. In MarvinSketch 16.4.18.0 entering Ac using the periodic table menu or keyboard short-cuts results in Actinium whilst using the “Label Editor” produces Acetyl. BIOVIA Draw 2017 makes a clear distinction when adding abbreviated atoms and both interpretations can be input. ChemDoodle 7.0.2 always interprets Ac as Actiniumwhen setting the atom label but does allow OAc for Acetoxy. With all of these, there is often little visual indication or feedback as to whether a user has entered the input they intended to.

To remove the ambiguity between Tosyl and Tennessine the alternative abbreviation Tos can be used. A brief analysis of sketches taken from United States patent applications published in 2015 shows that **Ts** is used in atom labels 2290 times and Tos 113 times.

## Case insensitivity

A more subtle problem may arise with the symbol **Nh** in software that allows case insensitive atom labels. It is reasonable to accept CL as equal to Cl for chlorine (e.g. PDB HETATMs) but NH (secondary amine) may now unexpectedly be picked up as Nihonium from the internal dictionary.

## SMARTS queries

Support for the SMARTS query language is available in many closed and open-source cheminformatics toolkits. A potential area for ambiguity is again found with Nihonium and the interpretation of other transfermium symbols. Transfermium symbols were officially named after the initial release of the Daylight SMARTS toolkit [5] and in subsequent implementations some are interpreted differently between toolkits either as an element or a conjuction (AND) expression. For example, at the time of writing both the CDK [6] and Open Babel [7] interpret [Bh] as [B&h] by whilst RDKit [8] interprets it as [#107].

The problem occurs due to the implicit conjunction between adjacent primitive expressions. The new symbol [Nh] could be interpreted as [#113] (element 113) or [N&h] (Aliphatic nitrogen and at least one implicit hydrogen). Table 1 lists the transfermium symbols and their different interpretations. Software that generates SMARTS patterns should take extra care to avoid writing ambiguous expressions.

**Table 1:** Ambiguous SMARTS for transfermium element symbols officially named since 1997

Ambiguous SMARTS	Element name	Element SMARTS	Expression meaning	Expression SMARTS
[No]	Nobelium	[#102]	Aliphatic nitrogen and aromatic oxygen (logically impossible)	–
[Db]	Dubnium	[#105]	Aromatic boron with on explicit bond (possible on fragment matching)	[D&b] or [bD]
[Bh]	Bohrium	[#107]	Aliphatic boron with at least one implicit hydrogen	[B&h] or [hB]
[Hs]	Hassium	[#108]	Aromatic sulfur with one explicit hydrogen	[H&s] or [sH]
[Ds]	Darmstadtium	[#110]	Aromatic sulfur with one explicit bond (possible on fragment matching)	[D&s] or [sD]
[Cn]	Copernicium	[#112]	Aliphatic Carbon and aromatic nitrogen (logically impossible)	–
[Nh]	Nihonium	[#113]	Nitrogen with at least one implicit hydrogen	[N&h] or [hN]

The meaning in SMARTS changes between cheminformatics toolkits and are either interpreted as matching specific elements or as expressions. Unambiguous SMARTS are provided for each of these cases

## Conclusions

A pragmatic approach to handling the new elements or perhaps all high atomic number elements with a very short half-life could be to simply ignore them. Whilst these elements are unlikely to have a practical application it is unsatisfactory to simply ignore them and we hope this commentary highlights that care should be taken when supporting the new symbols in cheminformatics software.
